# Study of a Natural Mutant SHV-Type **β**-Lactamase, SHV-104, from *Klebsiella pneumoniae*

**DOI:** 10.1155/2014/548656

**Published:** 2014-05-13

**Authors:** Nahed Ben Achour, Omrane Belhadj, Moreno Galleni, Mohamed Ben Moussa, Paola Sandra Mercuri

**Affiliations:** ^1^Laboratoire de Biochimie et de Technobiologie, Faculté des Sciences de Tunis, Campus Universitaire, EL Manar II, 2092 Tunis, Tunisia; ^2^Laboratoire de Macromolécules Biologiques, Centre d'Ingénierie des Protéines, Université de Liège, Sart-Tilman, 4000 Liège, Belgium; ^3^Service de Bactériologie, Hôpital Militaire de Tunis, Montfleury, 1008 Tunis, Tunisia

## Abstract

*Klebsiella pneumoniae* ML2011, a multiresistant isolate, was isolated from the Military Hospital of Tunis (Tunisia). The determination of the minimal inhibitory concentrations exhibited by *K. pneumoniae* ML2011 was performed by Etest. The crude extract of the isolates contains four different **β**-lactamases with pI 5.5, 7.3, 7.6, and 8.6. Only the **β**-lactamases with pI 7.3 and pI 8.6 were transferred by transformation and conjugation experiment. Molecular characterization of these genes was performed by PCR and sequencing. The chromosomal **β**-lactamases are TEM (pI 5.5) and SHV-1 (7.6). CTX-M-28 (pI 8.6) and the novel variant of SHV named SHV-104 (pI 7.3) were encoded by *bla* gene located on a 50 kb highly conjugative plasmid. The SHV-104 **β**-lactamase was produced in *E. coli* and purified. Its profile of activity was determined. Compared to SHV-1, SHV-104 contains one mutation, R202S. Their kinetic parameters were similar except for cefotaxime. The analysis of the predicted structure of SHV-104 indicated that the R202S mutation suppresses a salt bridge present in SHV-1. Therefore, the overall flexibility of the protein increased and might improve the hydrolysis of cefotaxime. We can conclude that the multiresistant phenotype of *K. pneumoniae* ML2011 strain is mainly linked to the production of CTX-M-28 since SHV-104 possesses a narrow spectrum of activity.

## 1. Introduction

*K. pneumoniae* belonging to the family of Enterobacteriaceae is an important opportunist human pathogen associated with hospital-acquired infections such as pneumonia, urinary tract infections, or bacteraemias [1–3]. This species naturally produces a broad-spectrum *β*-lactamase of the SHV-1 type or penicillinase LEN that confers resistance to penicillins and to first-generation cephalosporins [3]. The widespread use of expanded-spectrum antibiotics for treatment of serious infections due to Gram negative bacteria has led to the appearance of extended-spectrum *β*-lactamases (ESBLs) which are able to hydrolyze these compounds [4–8]. Since their first identification in Germany and the United States of America, extended-spectrum *β*-lactamases (ESBLs) have spread worldwide [6, 7]. These enzymes are mostly plasmid-encoded variants of TEM-1, TEM-2, and SHV-1 by one or more amino acids or are from a rapidly evolving class called CTX-M. They confer resistance to broad-spectrum penicillins, monobactams, and narrow-spectrum and third-generation cephalosporins, respectively [4, 8]. The prevalence of ESBL producing* K. pneumoniae* isolates in hospitals ranges from 5 to 25% in several parts of the world [7]. In September 2012, more than 100 SHV variants have been identified (http://www.lahey.org/; J. A. Jacoby and K. Bush). In Tunisia, SHV-2a and SHV-12 were the predominant SHV-type ESBL produced by* K. pneumoniae* [9].

In this work, we searched for the presence of potential new SHV's variants produced by* K. pneumoniae* isolates in Tunisia clinical settings. From this survey, we isolated a* K. pneumoniae* strain (ML2011) resistant to third-generation cephalosporin and aztreonam. This isolate produced a new SHV variant, SHV-104. This *β*-lactamase was produced and purified and its activity profile was characterized. Our goal was to study the contribution of SHV-104 in the multiresistant phenotype of the strain.

## 2. Materials and Methods

### 2.1. Bacterial Strains

*K. pneumoniae* ML2011 was collected in July 2004, from intensive care unit of the Military Hospital of Tunis (Tunisia). Identification of strains was performed by using both the VITEK automated system (bioMérieux, Marcy l'Etoile, France) and API 20 E system (bioMérieux, Marcy l'Etoile, France). Biochemical and serological confirmation of the identity of this strain was done at the Laboratory of Bacteriology, Military Hospital, Tunisia.* E. coli* DH5*α* and* E. coli* HB101 were used, respectively, for the transformation and conjugation experiments.* E. coli* BL21 and BL21 (DE3) (pLysS) were used for the resistance profile of the different strains and for the overexpression of SHV-104, respectively.

### 2.2. Antimicrobial Susceptibility Testing and Determination of Minimal Inhibitory Concentrations

Disc susceptibility testing was performed according to Clinical and Laboratory Standards Institute (CLSI) guidelines [11] on Mueller-Hinton agar (Bio Rad, France). Detection of ESBL activity was achieved with a double-disk synergy test, by screening isolate for synergism between amoxicillin-clavulanate and cefotaxime, ceftriaxone, ceftazidime, and aztreonam, respectively [12]. Minimal inhibitory concentrations (MICs) of ampicillin, ticarcillin, ceftriaxone, cefotaxime, ceftazidime, cefoxitin, imipenem, streptomycin, and chloramphenicol were determined by Etest strips, according to the manufacturer's instructions (AB Biodisk, Solna, Sweden), and interpreted according to clinical breakpoints from the CLSI and EUCAST.

### 2.3. Analytical Isoelectric Focusing (IEF)

pI of the *β*-lactamases produced by* K. pneumoniae* ML2011, the HB101 transconjugants, and the BL21 transformants were determined as described previously [13].

### 2.4. Plasmid Analysis and Transformation Experiments

Plasmid DNA of the clinical isolate was extracted with a plasmid extraction kit GFX Micro Plasmid Prep (Amersham Biosciences, UK), according to the manufacturer's instructions. Plasmid DNA electrophoresis was performed in 1% agarose gel and visualized with ethidium bromide under UV light. The plasmid DNA of* E. coli* RP4 (50 kb) was used as a molecular size marker [13, 14]. Transfer of the resistance determinant was performed with* E. coli* DH5*α* as the recipient. Transformants were selected on Luria Bertani (LB) medium agar plates supplemented with ampicillin (100 *μ*g/mL).

### 2.5. Conjugation Experiments

Transfer of resistance phenotypes was performed by a liquid mating method [9] on LB broth medium. Culture mixtures were incubated overnight at 37°C with transformants* E. coli* DH5*α*/pML2011 as donors and* E. coli* HB101 as recipient strain. After conjugation, bacterial suspensions were plated onto agar containing ampicillin (100 *μ*g/mL) and streptomycin (100 *μ*g/mL). The resulting transconjugants were purified and identified with API 20 E strips.

### 2.6. Polymerase Chain Reaction (PCR)

Amplifications of the *bla*_TEM_, *bla*_SHV_, and *bla*_CTX-M_ genes by PCR were carried out using the plasmid pML2011 and the chromosomal DNA of* K. pneumoniae* ML2011 as the template. The PCR was performed as described previously in [15], Table 1.

### 2.7. Sequencing of PCR Product

The PCR products (corresponding to SHV and CTX-M genes) were visualized by agarose gel electrophoresis, purified with a GFX PCR DNA and Gel Band Purification kit (Amersham Biosciences, UK). Prior to their cloning into pTZ57R/T vector (Fermentas, USA), the sequence of the PCR fragment was verified by GIGA Genomics Facility (Ulg, Belgium). The ligation mix was transformed into* E. coli* DH5*α* competent cells. Positive colonies were selected on LB broth medium supplemented with ampicillin (100 *μ*g/mL). The DNA plasmids were extracted with GeneJet Plasmid Miniprep (Fermentas, USA). The insert sequences were performed by GIGA Genomics Facility in Belgium, using forward and reverse M13 universal primers: PUC/M13F (5′-CGC CAG GGT TTT CCC AGT CAC GAC-3′) and PUC/M13 R (5′-TCA CAC AGG AAA CAG CTA TGA C-3′).

### 2.8. Expression and Purification of SHV-104

*Nde*I and* Bam*HI restriction sites were inserted at the 5′ and 3′ extremities of *bla*_SHV_, respectively, by PCR. The nucleotides sequences of the PCR-generated fragments were firstly verified by sequencing and secondly cloned into the pET26b (+) vector (Novagen Inc., Madison, Wis, USA). The corresponding vector, pET26b/SHV-104, was used to transform* E. coli* BL21 (DE3) (pLysS) competent cells (Novagen Inc., Madison, Wis, USA). The SHV-104 enzyme was produced in Terrific Broth (TB) medium containing kanamycin (50 *μ*g/mL) and chloramphenicol (30 *μ*g/mL) as selecting agents during growth of the bacteria at 37°C under orbital shaking. 40 mL of an overnight preculture in TB was inoculated to 1 L of fresh TB supplemented with kanamycin and chloramphenicol. At an absorbance value of 0.7 at 600 nm, IPTG (final concentration 0.5 mM) was added and the culture was incubated for 5 additional hours. Cells were harvested by centrifugation (5,000 g for 10 min at 4°C); the pellet was resuspended in 50 mL of 20 mM Tris/HCl buffer pH 7.5 (buffer A). Bacteria were disrupted with the help of cell disrupter equipment (Emulsiflex C3, Germany). Cell debris were removed by centrifugation (30 000 g for 45 min at 4°C) and the supernatant was dialysed overnight against buffer A at 4°C. The crude extract was loaded onto a Q-Sepharose FF column (2.6 by 34 cm, Pharmacia, Sweden) equilibrated in buffer A. The *β*-lactamase SHV-104 was eluted in the flow through. The solution was dialysed overnight against 20 mM Tris/HCl buffer pH 8.5 and loaded in a HiTrap Q-Sepharose HP (5 mL; GE Healtcare). The enzyme was eluted by a linear NaCl gradient (0–0.7 M) in ten column volumes. The active fractions were collected and concentrated on YM-10 membrane (Amicon, Beverly, Mass.) to a final volume of 3 mL. The sample was loaded in a molecular sieve Sephacryl-100 (1.5 × 56 cm) column equilibrated in 25 mM sodium phosphate buffer pH 7.0 containing 0.2 M NaCl. The fractions exhibiting *β*-lactamase activity were collected and their specific activity was followed by measuring the rate of nitrocefin hydrolysis. The fractions exhibiting a constant specific activity were pooled and concentrated to a final concentration of 0.5 mg/mL. The enzyme preparation was stored at −20°C in 25 mM sodium phosphate buffer pH 7.0. The N-terminal sequence was determined with the help of a gas-phase sequencer (Prosite 492; Applied Biosystems, Calif).

### 2.9. Determination of Kinetic Parameters

All the measurements were done on a spectrophotometer Specord 50 analytik jena (Analis, Belgium) connected to a personal computer via an RS232C interface. The reactions were performed in a total volume of 500 *μ*L at 30°C. Bovine serum albumin (20 *μ*g/mL) was added to diluted solutions of *β*-lactamase in order to prevent enzyme denaturation. The steady state kinetic parameters (Km and *k*_cat_) were determined using the Hanes' linearization of the Michaelis-Menten equation. The *k*_cat_ and Km values were determined for a representative set of *β*-lactam antibiotics.

### 2.10. Molecular Modeling

A model of the SHV-104 structure was built with the help of Easypred program [16] using SHV-1 as template.

## 3. Results and Discussion

### 3.1. Antimicrobial Susceptibility Testing

MICs for* K. pneumoniae* ML2011 showed that this strain was resistant to all *β*-lactams tested except imipenem (Table 2).* K. pneumoniae* ML20011 exhibited a high level of resistance to oxyimino-cephalosporins (cefotaxime, ceftriaxone, and cefpirome: MIC 512 mg/L; ceftazidime: MIC 256 mg/L). The strain was also resistant to chloramphenicol, ciprofloxacin, nalidixic acid, and tetracycline. The disk diffusion method performed according to the CLSI guidelines [11] showed synergistic effect between ceftazidime, cefotaxime, aztreonam, ceftriaxone, and amoxicillin-clavulanic acid suggesting the presence of an ESBL enzyme [12].

### 3.2. Plasmid Analysis and Transfer of Resistance

Plasmid analysis revealed the presence of a 50 kb transferable plasmid by transformation and conjugation experiments. This plasmid was named pML2011. The frequency of conjugational transfer performed with* K. pneumoniae* ML2011 as donors and* E. coli* HB101 as the recipient was 10^−2^/donor. The* E. coli* transformants and transconjugants were resistant to penicillins and expanded-spectrum cephalosporins but were susceptible to cefoxitin, aminoglycosides, quinolones, chloramphenicol, and imipenem (Table 2).

### 3.3. Analytical Isoelectric Focusing (IEF)

The examination of the crude extract of* K. pneumoniae* ML2011 by isoelectric focusing showed the presence of four *β*-lactamase bands with apparent pIs of 5.5, 7.3, 7.6, and 8.6. Only the *β*-lactamases with pI 8.6 and 7.3 were transferred by transformation and conjugation experiments.

### 3.4. PCR Amplifications and Sequencing Analysis

PCR analyses confirmed the presence of *bla*_TEM_, *bla*_CTX-M-1_ group, and *bla*_SHV_ in* K. pneumoniae* ML2011. *bla*_SHV_ can be present on both the chromosomal DNA of* K. pneumoniae* ML2011 and the plasmid pML2011. *bla*_CTX-M_ was detected only in the plasmid pML2011. The complete nucleotide sequences were performed and compared for SHV gene with strain* K. pneumoniae* Kp297 (DDBJ/EMBL/GeneBank accession number EF035567) and CTX-M gene with CTX-M-1 GeneBank accession number X92506. These comparisons revealed the presence of CTX-M-28 previously described in [13] and 2 SHV: *bla*_SHV-1_ on the chromosomal DNA and an open reading frame that was similar to *bla*_SHV-1_ with one mutation on the plasmid pML2011. The mutation consisted of the replacement of the (R) residue at position 202 (codon CGT) to S (codon AGT). As this substitution was not present in all known SHV *β*-lactamases, the sequence of *bla*_SHV_ was named *bla*_SHV-104_ and was deposited in GenBank (accession number EU274581). The analysis of the SHV sequences coding mutation at amino acid 202 suggests that SHV-104 has evolved directly from SHV-1. Moreover, the modification R202S may result in a change of the pI to 7.3.

### 3.5. Purification and Characterization of SHV-104

The best results, in terms of production of SHV-104, were found in growing* E. coli* BL21(DE3) (pLysS) in TB, added 0.5 mM IPTG after 5-hour incubation. The SHV-104 was extracted from the periplasmic space and purified by three chromatographic steps (Q-Sepharose FF, HiTrap Q-Sepharose HP, and molecular sieve Sephacryl-100) to obtain at the end ~10 mg (90% protein pure) for liter. The resulting N-terminal sequence of the purified SHV-104 was MRYIRLCIISL as expected. Kinetic parameters values, *k*_cat_ and Km, of SHV-104 *β*-lactamase for representative *β*-lactam antibiotics were shown in Table 3. *k*_cat_ values of SHV-104 against ticarcillin, nitrocefin, cephalothin, and cefuroxime were higher than those of SHV-1, and Km values were higher (43 to 540 *μ*M versus 21 to 100 *μ*M) except for ticarcillin. Overall, the catalytic efficiency against penicillins was higher for SHV-104 (*k*_cat_/Km, 0.001 to 19 *μ*M^−1^ s^−1^) than for SHV-1 (*k*_cat_/Km, 0.3 to 14 *μ*M^−1^ s^−1^), except for cefuroxime (*k*_cat_/Km, 0.001 *μ*M^−1^ s^−1^ versus 0.005 *μ*M^−1^ s^−1^). In contrast, SHV-104 had a low catalytic efficiency against benzylpenicillin (30-fold) than SHV-1 and exhibited catalytic activity against cefotaxime (an oxyimino-cephalosporin).

Kinetic analysis showed that SHV-104 was more active against nitrocefin and cephalothin, but hydrolysis remained inefficient, owing to a high Km. Ticarcillin was better hydrolyzed by SHV-104 than SHV-1. Among oxyimino-cephalosporins, only cefotaxime was slowly hydrolysed by SHV-104. On the basis of the fact that a group 2be enzyme should hydrolyze one or more oxyimino-*β*-lactams such as cefotaxime, ceftazidime, or aztreonam at 10% the rate at which benzylpenicillin is hydrolyzed [17, 18], the activity of the novel enzyme was insufficient for the enzyme to count as an extended-spectrum *β*-lactamase (ESBL).

### 3.6. Molecular Modeling of SHV-104 Enzyme

**Figure 1:**
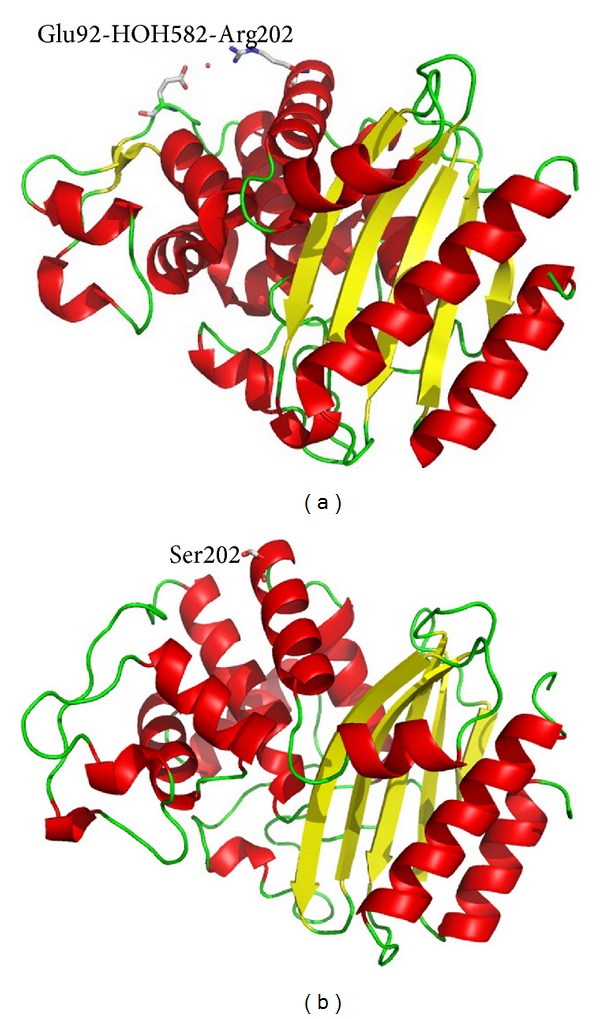
Tertiary structure of (a) SHV-1 and (b) SHV-104. The *β*-strands are shown in yellow and the helices *α* in red.

Finally, a model of the SHV-104 structure was built with the help of Easypred program using SHV-1 as template. The R202S mutation may affect a salt bridge present in SHV-1 between R202 and E92 via a water molecule (Figure 1). Analysis of SHV-104 structure model showed that it is reasonable to think that this mutation cannot have a direct effect on the broadening of the activity profile of the SHV enzyme. A plausible hypothesis relies on the fact that the absence of a salt bridge in SHV-104 compared to SHV-1 could increase the overall flexibility of the protein and indirectly the flexibility of the *β*-lactamase active site. This phenomenon may allow a better interaction and hydrolysis of cefotaxime by SHV-104 variant. The conversion of a non-ESBL to an ESBL for the SHV family can be achieved by different mutations [19]. The G238S and E240K substitutions allow the expansion of the SHV's activity profiles. Unlike the earliest described ESBLs (SHV-4, -5, -7, -9, -10, -12, -15, -22, -45, -46, -55, -64, and -66), SHV-104 does not possess these mutations, G238S and E240K, thought initially to be necessary for the hydrolysis of cefotaxime. Mutation at position 179 was responsible for the ESBL phenotype in SHV-6, -8, and -24. The N179G mutation confers resistance to ceftazidime and ceftriaxone [20]. Random mutagenesis of 179 in TEM-1 produced three* E. coli* transformants (179N, G, and Y) with increased levels of resistance to ceftazidime [21]. SHV-7 variant was found with a serine at position 43 and is resistant to cefotaxime, ceftazidime, and aztreonam. The result of a change at position 43 is unclear. The hydrogen bonding from R43 to the segment at positions 64 to 69 running behind the *β*-sheet may be important in maintaining the position of S70 [21].

In conclusion, we could demonstrate that the production of SHV-104 is not the major factor for the resistance pattern of* K. pneumonia* ML2011. Our study indicates that SHV-104 can be an intermediate in the process of selection of a new SHV boras spectrum *β*-lactamase. We could also conclude that the coexpression of CTXM-28 by this strain will increase the resistance profile of the strain toward beta-lactam antibiotics.

## Figures and Tables

**Table 1:** Sequences of the primers used to detect *β*-lactamases genes.

PCR target	Primer name	Primer sequence	Annealing temperatures on °C	Amplicon sizes
CTX-M-1 group	CTX-1F	5′ATGGTTAAAAAATCACTGCGTC3′	60	864 pb
	CTX-1R	5′TTGGTGACGATTTTAGCCGC3′	60	

CTX-M-2 group	CTX-2F	5′ATGATGACTCAGAGCATTCG3′	58	866 pb
	CTX-2R	5′TGGGTTACGATTTTCGCCGC3′	62	

CTX-M-8 group	CTX-8F	5′ACTTCAGCCACACGGATTCA3′	60	877 pb
	CTX-8R	5′CGAGTACGTCACGACGACTT3′	62	

CTX-M-9 group	CTX-9F	5′ATGGTGACAAAGAGAGTGCAA3′	60	876 pb
	CTX-9R	5′TCACAGCCCTTCGGCGATGATTCTCGC3′	86	

SHV	SHV-1F	5′ATGCGTTATATTCGCCTGTGTATT3′	66	868 pb
	SHV-1R	5′-TTAGCGTTGCCAGTGCTCGATCAG-3′	74	

TEM	TEM-1F	5′ATAAAATTCTTGAAGACGAAA3′	52	1080 pb
	TEM-1R	5′GACAGTTACCAATGCTTAATC3′	58	

**Table 2:** MICs of various antimicrobial agents obtained for the clinical isolate *K.  pneumoniae* ML2011, transformants, transconjugants, and the *E. coli* recipients.

Antibiotics	*K. pneumoniae *	*E. coli *					
	pML2011	HB 101	HB 101 × pML2011	DH5*α*	DH5*α*/pML2011	BL21/pET26b	BL21/pET26b *bla*_SHV-104_
Ampicillin	>512	4	>512	8	>512	8	>512
Amoxicillin	>512	4	>512	8	>512	8	>512
Ticarcillin	>512	2	>512	2	>512	2	>512
Cephaloridine	>512	1	>512	0.13	>512	ND	ND
Cefoxitin	64	2	8	4	8	2	2
Cefotaxime	>512	1	512	0.13	512	2	8
Ceftazidime	256	1	64	0.13	64	2	2
Ceftriaxone	>512	1	512	0.13	512	2	2
Cefpirome	>512	2	>512	0.13	>512	ND	ND
Aztreonam	256	1	256	0.13	256	ND	ND
Imipenem	2	0.25	0.38	0.06	0.38	ND	ND
Chloramphenicol	>256	2	2	2	2	4	4
Nalidixic acid	>512	1	1	0.06	1	1	1
Ciprofloxacin	256	1	1	0.06	1	ND	ND
Tetracycline	>512	2	2	0.25	2	ND	ND

ND: not determinable.

**Table 3:** Kinetics parameters.

Antibiotics	SHV-104			SHV-1		
	*k*_cat_	Km	*k*_cat_/Km	*k*_cat_	Km	*k*_cat_/Km
	(sec^−1^)	(*μ*M)	(*μ*M^−1^ sec^−1^)	(sec^−1^)	(*μ*M)	(*μ*M^−1^ sec^−1^)
Benzylpenicillin	55	94	0.6	360	20	18
Ticarcillin	80	10	8	80	27	3
Nitrocefin	830	43	19	290^a^	21^a^	14^a^
Cephalothin	30	68	0.44	9	30	0.3
Cefuroxime	0.53	540	0.001	0.5	100	0.005
Cefotaxime	>1.8	>600	0.003	N.D.	N.D.	N.D.

ND: not determinable because the hydrolysis rate was too low.

^a^The kinetic constants of nitrocefin for SHV-1 are from Thomson et al. [10].
